# [^**11**^C]-Methionine Positron Emission Tomography in the Postoperative Imaging and Followup of Patients with Primary and Recurrent Gliomas

**DOI:** 10.1155/2014/463152

**Published:** 2014-02-04

**Authors:** Matteo Santoni, Cristina Nanni, Alessandro Bittoni, Gabriele Polonara, Alessandro Paccapelo, Roberto Trignani, Mariagrazia De Lisa, Franco Rychlicki, Luciano Burattini, Rossana Berardi, Stefano Fanti, Stefano Cascinu

**Affiliations:** ^1^Dipartimento di Oncologia Medica, Università Politecnica delle Marche, AOU Ospedali Riuniti, 60126 Ancona, Italy; ^2^Dipartimento di Medicina Nucleare, Azienda Ospedaliero-Universitaria S. Orsola-Malpighi, 40125 Bologna, Italy; ^3^Dipartimento di Neuroradiologia, Università Politecnica delle Marche, AOU Ospedali Riuniti, 60126 Ancona, Italy; ^4^Divisione di Neurochirurgia, Università Politecnica delle Marche, AOU Ospedali Riuniti, 60126 Ancona, Italy

## Abstract

We investigated the sensitivity and specificity of [^11^C]-methionine positron emission tomography ([^11^C]-MET PET) in the management of glioma patients. We retrospectively analysed data from 53 patients with primary gliomas (16 low grade astrocytomas, 15 anaplastic astrocytomas and 22 glioblastomas) and Karnofsky Performance Status (KPS) > 70. Patients underwent [^11^C]-MET PET scans (*N* = 249) and parallel contrast-enhanced MRI (*N* = 193) and/or CT (*N* = 113) controls. In low grade glioma patients, MRI or CT findings associated with [^11^C]-MET PET additional data allowed discrimination residual disease from postsurgical changes in 96.22% of these cases. [^11^C]-MET PET early allowed detection of malignant progression from low grade to anaplastic astrocytoma with high sensitivity (91.56%) and specificity (95.18%). In anaplastic astrocytomas, we registered high sensitivity (93.97%) and specificity (95.18%) in the postoperative imaging and during the followup of these patients. In GBM patients, CT and/or MRI scans with additional [^11^C]-MET PET data registered a sensitivity of 96.92% in the postsurgical evaluation and in the tumour assessment during temozolomide therapy. A significant correlation was found between [^11^C]-MET mean uptake index and histologic grading (*P* < 0.001). These findings support the notion that complementary information derived from [^11^C]-MET PET may be helpful in postoperative and successive tumor assessment of glioma patients.

## 1. Introduction

The therapeutic approach to glioma patients remains a major challenge in clinical neurooncology, mainly for the inefficiency of the conventional instrumental methods to assess the response to the different therapeutic tools. In fact, an adequate imaging may affect the assessment of an objective response rate and the determination of the progression-free survival, thus influencing the therapeutic course of glioma patients.

In neuroradiology, contrast enhancement reflects blood-brain barrier damage regions and it is considered an indirect marker for active tumor tissue. However, MRI contrast enhancement often does not adequately differentiate viable tumor tissue from necrosis induced by therapy or occurring spontaneously during tumour progression [1]. Furthermore, contrast enhancement may be confused by dexamethasone, which reduces the uptake of contrast agents [2].

Positron emission tomography (PET) may improve the management of malignant glioma by identifying areas of increased metabolic activity beyond the area of contrast enhancement on MRI, which may correspond to the areas at highest risk of recurrence. Moreover, PET imaging may afford an improvement in the outline of radiation target volumes and in the assessment of responses to therapy.

In PET comparative studies on metabolic assessment of gliomas using different tracers show that MET-PET presents a higher efficacy than [18F]-fluorodeoxyglucose (FDG)-PET and [^11^C]-choline (CHO)-PET in the detection of hot lesions [3].

The aim of our study was to assess the sensivity and specificity of [^11^C]-MET PET to detect malignant progression from low grade to anaplastic astrocytoma and in the tumor response assessment to temozolomide (TMZ) therapy for anaplastic astrocytoma and glioblastoma patients of [^11^C]-MET PET, in addition to standard diagnostic imaging techniques, such as CT scan or MRI.

## 2. Materials and Methods

The study population consisted of adults (aged 18 years and above) with primary gliomas and Karnofsky Performance Status (KPS) > 70. Between January 2006 and June 2011, patients underwent [^11^C]-MET PET controls in our institutions. Data were retrospectively collected from patients, electronic medical records and paper charts. [^11^C]-MET PET controls were performed within one month from surgical resection and during their followup, with parallel MRI and CT scans. Patients with glioblastoma were followed from the postoperative setting to the progression on Stupp regimen [4].

The mean time period between serial controls was 6 months for low grade astrocytoma and 3 months for anaplastic astrocytoma and glioblastoma patients. Tumors were graded according to the World Health Organization (WHO) classification for neuroepithelial tumors [5].

Standard Macdonald criteria were used to determine response to chemoradiation [6]. Statistical analysis was performed with MedCalc—version 11.4.4.0 (MedCalc Software, Broekstraat 52, 9030 Mariakerke, Belgium). Overall Survival (OS) was analyzed using the Kaplan-Meier method [7].

All the patients performed a [^11^C]-methionine PET/CT using a 3D PET/CT scanner (GE, Discovery STE). They were IV injected with a mean dose of 210 MBq of tracer and the uptake time was 20 minutes. Fasting and specific prior analysis were not required for [^11^C]-MET PET/CT since collateral effects were never reported. PET images were acquired in 3D mode for 5 minutes per bed position (Field of View 15.7 cm, 47 slices) and reconstructed using a fully 3D iterative algorithm (ViewPoint algorithm, 2 iterations, 20 subsets). Attenuation correction was based on low-dose CT. The in-plane spatial resolution of the scanner was 5.3 mm. The parameters of the multidetector helical CT scan were 120 kV, 80 mA, 0.8 s per tube rotation, slice thickness of 3.75 mm, pitch of 1.675 : 1, and table speed of 33.5 mm/rot. CT images were used for both attenuation correction of emission data and image fusion. The relative index of [^11^C]-MET uptake was calculated as the ratio of tumor area to reference area.

CT scans were acquired with a spiral scanner General Electric Prospeed SX. Section orientation was parallel to the orbitomeatal line. Brain sections (time of 2 s, 120 kV, 160–200 mA, section thickness of 5 mm, and no intersection gap) were taken from the inferior aspect of the cerebellum to the vertex of the cranium. All scanning was set to a matrix of 512 × 512. An Ionidate contrast agent was used to better study brain lesions with blood-brain barrier breakdown.

MR images were acquired on a 1.5T MR-imaging scanner (Signa Excite HDxt; GE Healthcare, Milwaukee, Wisconsin) by using the body coil for transmission and an 8-channel receive coil array for signal-intensity detection. A morphological examination of the brain was obtained in all patients using 3–5 mm thick axial and coronal FLAIR and FSE T2-w sequences and SE T1-weighted images before and after paramagnetic contrast media injection. Diffusion, MR perfusion, and MR spectroscopy were also performed to obtain some vascular and metabolic informations.

All the areas presenting an increased tracer uptake above the background were considered positive. Standardized uptake value (SUV) max (normalized on the body weight) was calculated lesion by lesion.

Significance level for all analyses was set at a 0.05 level for all tests and all *P* values were two-sided. Comparison of [^11^C]-MET mean uptake index between histological groups was performed via Kruskal-Wallis nonparametric test. Statistical analysis was conducted with R-Software version 3.0.1 (The R Company, Vienna, Austria).

## 3. Results

We retrospectively analysed data from 53 consecutive patients with primary gliomas (16 low grade astrocytomas; 15 anaplastic astrocytoma; 22 glioblastomas) and KPS > 70 followed in our institutions between January 2006 and June 2011. Median age was 54 y (18–71). The majority of them had Karnofsky Performance Status (KPS) > 80. Patient characteristics are listed in Table 1.

These patients underwent [^11^C]-MET PET scans (*N* = 249) and parallel contrast-enhanced MRI (*N* = 193) and/or CT (*N* = 113) controls. A median of 4 PET scans (range 1–12) was performed for each patient.

**Figure 1:**
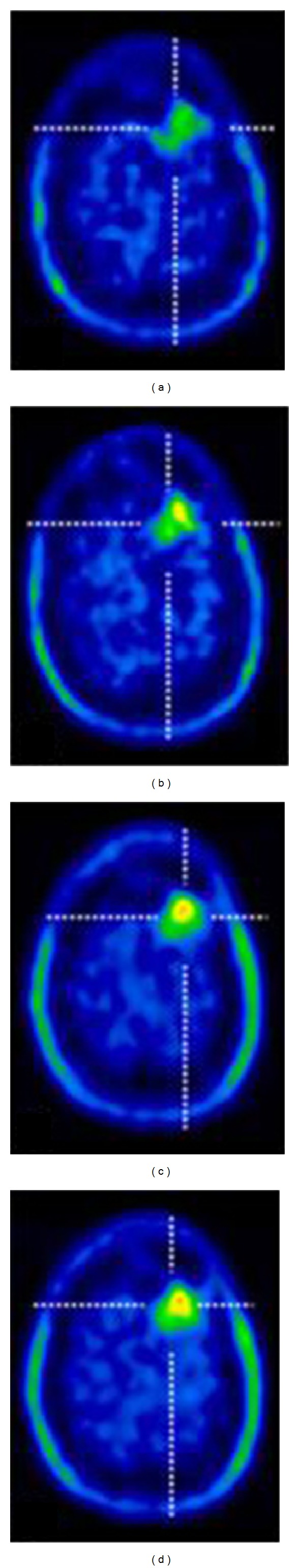
((a)–(d))**** ^11^C-MET PET image of a 44-year-old man with a frontal-parasagittal low grade glioma, after 1 month from surgical resection ((a)-(b)) and 3 months later ((c)-(d)), showing residual disease in the area of surgery.

**Figure 2:**
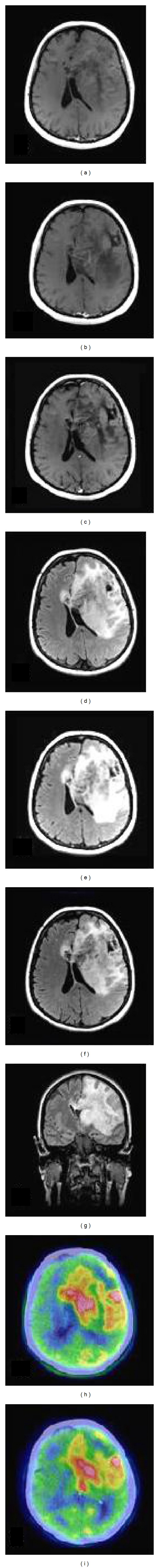
[^11^C]-MET PET and MRI scans of 54-year-old woman with malignant progression of grade II astrocytoma. On the left ((a), (d), and (g)), newly diagnosed left frontal-insular astrocytoma WHO grade II infiltrating the anterior portion of the corpus callosum. In the center ((b), (e), and (h)) One year later, MRI scans show increased tumor extension and compression on the left ventricular, associated with a significant increase in [^11^C]-MET uptake, expressing malignant progression to grade III astrocytoma. On this basis, we decided to treat this patient with TMZ 200 mg/m^2^ daily × 5 every 28 days. On the right ((c), (f), and (i)), three months after starting treatment with TMZ. MRI scans and [^11^C]-MET PET reveal a reduction of tumor extension and compression on the left ventricular, associated with a decreased [^11^C]-MET uptake.

Sixteen patients with low grade astrocytomas performed 84 [^11^C]-MET PET scans with corresponding MRI or CT controls. In 67.92% of postsurgical evaluations, MRI or CT findings alone allowed discrimination of residual disease from postsurgical treatment changes, with a 28.30% worked out by [^11^C]-MET PET additional data (Figure 1). Furthermore, [^11^C]-MET PET early allowed detection of malignant progression from low grade astrocytoma to anaplastic astrocytoma. Thus, in the 84 scans performed during their followup, we reported a sensitivity of 91.56% and a specificity of 95.18% in the detection of malignant progression.  Figure 2 shows the efficacy of [^11^C] MET PET in distinguishing malignant progression from grade II to grade III astrocytoma and in assessing tumour response three months after starting treatment with TMZ.

Similarly, we analyzed [^11^C]-MET PET in 15 patients with anaplastic astrocytomas, subjected to 90 [^11^C]-MET PET scans corresponding to MRI or CT controls. As observer in the followup of low grade astrocytoma patients, we found a high sensitivity (93.97%) and specificity (95.18%) in the postoperative evaluation of anaplastic astrocytoma patients, with four false-negative results. In 73.33% of postsurgical assessments, MRI or CT permitted differentiation residual disease from postsurgical changes, with a 23.67% assessed by the contribution of data from [^11^C]-MET PET scans.

Finally, we analyzed [^11^C]-MET PET employment in 22 patients with a histological diagnosis of GBM, treated with prior surgery and concomitant radiochemotherapy and adjuvant TMZ. Patients underwent serial [^11^C]-MET PET scans (*N* = 65) with corresponding contrast-enhanced MRI (*N* = 53) and/or CT (*N* = 65) scans. In this group, CT and/or MRI scans with additional [^11^C]-MET PET data registered a sensitivity of 96.92% in the postsurgical evaluation and in the tumour assessment during TMZ therapy, with no false-positive [^11^C]-MET uptake findings.

**Figure 3:**
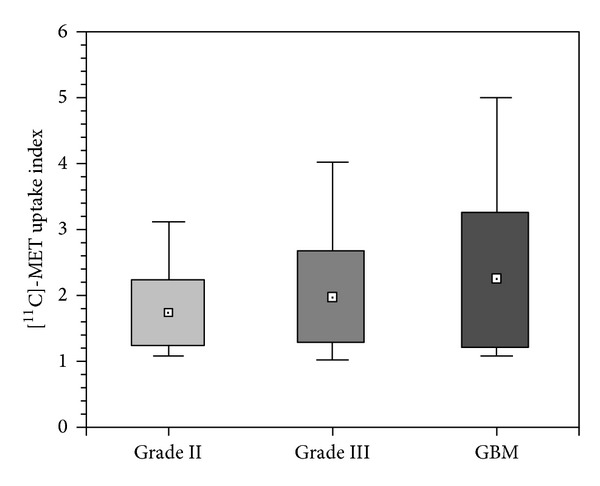
Tumor uptake index of [^11^C]-MET stratified on histological basis as indicated in the text. Boxes show 1 SD from the mean value, represented as a plot box, and whiskers show the minimal and maximum values for each group.

Moreover, we analyzed the [^11^C]-MET uptake index distribution in the three histological groups. Mean uptake index was, respectively, 1.73 (CI: 1.34–2.12; range: 1.13–3.14) for low grade astrocytomas, 1.99 (CI: 1.47–2.50; range: 1.06–4.00) for anaplastic astrocytomas, and 2.24 (CI: 1.71–2.78; range: 1.16–5.00) for GBMs. Based on these data, we analysed via Kruskal-Wallis nonparametric test the correlation between [^11^C]-MET mean uptake index and histological grading, whose results are absolutely significant (*P* = 0.0009).  Figure 3 shows the distribution of uptake index in the three groups of patients. No relation was found between the [^11^C]-MET mean uptake index and the OS of these patients (*P* = 0.15).

## 4. Discussion

Morphological imaging often does not adequately reflect tumour biology and its metabolic activity. [^11^C]-MET PET may afford additional information need in order to define better the biology of tumors and their response to treatment. Biological data, integrated with CT or MRI anatomical data, may acquire clinical significance in response assessment, in recurrence detection, and in the differentiation of postsurgical or radiation effects and tumor recurrence. Interestingly, the uptake of [^11^C]-methionine correlates with Ki-67 [8] and proliferating cell nuclear antigen expression and with microvessel count in proliferating cells [9], suggesting that [^11^C]-MET uptake may represent a biological marker for tumour proliferation and neoangiogenesis.

In 1989, Derlon et al. reported their preliminary experience about the regional cerebral methionine uptake with PET after intravenous injection of [^11^C]L-methionine. They correlated the ratio *R* between symmetrical regions of interest with the histological grading of gliomas [10]. Similarly, in 2003, Kracht et al. observed that [^11^C]-methionine uptake was significantly higher in high-grade gliomas as compared to low-grade gliomas [11], even if [^11^C]-MET uptake allows the identification of low grade gliomas. Previous studies investigated the efficacy of [^11^C]-MET PET in the identification of residual tumor after resection and recurrent gliomas [12] and in the malignant transformation of low grade gliomas. Additionally, the volumetric assessment of [^11^C]-MET PET resulted frequently wider than assessed by MRI or CT, sustaining the hypothesis that [^11^C]-MET PET permits the detection of subareas of active tumour volume in addition to those observed by MRI or CT enhancement alone [13–15]. This event is even more frequent in low-grade tumors and in diffuse gliomatosis depending on their frequent lack of contrast enhancement in MRI [16].

Recently, Galldiks et al. reported that pretreatment volumetry of MET uptake but not the semiquantitative MET uptake ratio is a useful biologic prognostic marker in patients with malignant glioma [17]. Furthermore, Matsuo et al. have investigated the role of [^11^C]-MET PET in the definition of GBM in radiation therapy planning [18]. Thirty-two patients with newly GBM performed CT, MRI, and [^11^C]-MET PET within two weeks after undergoing surgery. The results indicate that [^11^C]-MET PET has a substantial impact on detecting the extent of GBM, allowing a more precise delineation of target volumes in radiation therapy planning.

Furthermore, Terakawa et al. published a study on seventy-seven patients who had been previously treated with radiotherapy after primary treatment for metastatic brain tumor or glioma. They showed that [^11^C]-MET PET can provide quantitative values to aid in the differentiation of tumor recurrence from radiation necrosis [19].

In our study population, [^11^C]-MET PET associated with CT and/or MRI scans enabled the assessment of postsurgery status in both low and anaplastic astrocytomas and the early detection of malignant progression in low grade astrocytoma patients with high sensition and specificity, compared with MRI and CT findings alone. Furthermore, [^11^C]-MET PET expressed its maximum potential in the postoperative imaging and in earlier disclosing recurrence of anaplastic astrocytoma and GBM patients treated with TMZ, offering more reliable assessment of treatment and allowing refining individualized treatment strategies. The potential clinical impact of earlier detecting tumor progression of anaplastic astrocytoma and GBM patients on their outcome should be investigated in randomized trials. Our findings support the notion that complementary information derived from [^11^C]-MET uptake may be helpful to diagnose and define tumor activity and finally to assess tumor response in patients with primary and recurrent gliomas.

## Figures and Tables

**Table 1:** Patient characteristics.

Gender	
Female	27
Male	26
Age, years	
Median	54
Range	18–71
Karnofsky Performance Status	
70–80	15
90–100	38
Histology	
Low grade glioma	16
Anaplastic astrocytoma	15
Glioblastoma multiforme	22
Surgery	
Undergone	44
Not undergone	9
Number of [^11^C]-MET PET	
Median	4
Range	2–12

## References

[1] van den Bent MJ, Vogelbaum MA, Wen PY, Macdonald DR, Chang SM (2009). End point assessment in gliomas: novel treatments limit usefulness of classical Macdonald’s criteria. *Journal of Clinical Oncology*.

[2] ØStergaard L, Hochberg FH, Rabinov JD (1999). Early changes measured by magnetic resonance imaging in cerebral blood flow, blood volume, and blood-brain barrier permeability following dexamethasone treatment in patients with brain tumors. *Journal of Neurosurgery*.

[3] Kato T, Shinoda J, Nakayama N (2008). Metabolic assessment of gliomas using ^11^C-methionine, [^18^F] fluorodeoxyglucose, and ^11^C-choline positron-emission tomography. *American Journal of Neuroradiology*.

[4] Stupp R, Hegi ME, Mason WP (2009). Effects of radiotherapy with concomitant and adjuvant temozolomide versus radiotherapy alone on survival in glioblastoma in a randomised phase III study: 5-year analysis of the EORTC-NCIC trial. *The Lancet Oncology*.

[5] Louis DN, Ohgaki H, Wiestler OD (2007). The 2007 WHO classification of tumours of the central nervous system. *Acta Neuropathologica*.

[6] Macdonald DR, Cascino TL, Schold SC, Cairncross JG (1990). Response criteria for phase II studies of supratentorial malignant glioma. *Journal of Clinical Oncology*.

[7] Kaplan E, Meier P (1958). Non parametric estimation for incomplete observation. *Journal of the American Statistical Association*.

[8] Chung JK, Kim Y, Kim SK (2002). Usefulness of ^11^C-methionine PET in the evaluation of brain lesions that are hypo- or isometabolic on ^18^F-FDG PET. *European Journal of Nuclear Medicine*.

[9] Sato N, Suzuki M, Kuwata N (1999). Evaluation of the malignancy of glioma using ^11^C-methionine positron emission tomography and proliferating cell nuclear antigen staining. *Neurosurgical Review*.

[10] Derlon JM, Bourdet C, Bustany P (1989). [^11^C]L-Methionine uptake in gliomas. *Neurosurgery*.

[11] Kracht LW, Friese M, Herholz K (2003). Methyl-[^11^C]-L-methionine uptake as measured by positron emission tomography correlates to microvessel density in patients with glioma. *European Journal of Nuclear Medicine and Molecular Imaging*.

[12] Thornton AF, Hegarty TJ, Ten HRK (1991). Three dimensional treatment planning of astrocytomas: a dosimetry study of cerebral irradiation. *International Journal of Radiation Oncology ∗ Biology ∗ Physics*.

[13] Jacobs AH, Winkeler A, Dittmar C (2002). Molecular and functional imaging technology for the development of efficient treatment strategies for gliomas. *Technology in Cancer Research and Treatment*.

[14] Grosu AL, Weber WA, Riedel E (2005). L-(methyl-^11^C) methionine positron emission tomography for target delineation in resected high-grade gliomas before radiotherapy. *International Journal of Radiation Oncology Biology Physics*.

[15] Ogawa T, Kanno I, Shishido F (1991). Clinical value of PET with ^18^F-fluorodeoxyglucose and L-methyl-^11^C-methionine for diagnosis of recurrent brain tumor and radiation injury. *Acta Radiologica*.

[16] Mineura K, Sasajima T, Kowada M, Uesaka Y, Shishido F (1991). Innovative approach in the diagnosis of gliomatosis cerebri using carbon-11-L-methionine positron emission tomography. *Journal of Nuclear Medicine*.

[17] Galldiks N, Dunkl V, Kracht LW (2012). Volumetry of [*¹¹*C]-methionine positron emission tomographic uptake as a prognostic marker before treatment of patients with malignant glioma. *Molecular Imaging*.

[18] Matsuo M, Miwa K, Tanaka O (2012). Impact of [^11^C]methionine positron emission tomography for target definition of glioblastoma multiforme in radiation therapy planning. *International Journal of Radiation Oncology Biology Physics*.

[19] Terakawa Y, Tsuyuguchi N, Iwai Y (2008). Diagnostic accuracy of ^11^C-methionine PET for differentiation of recurrent brain tumors from radiation necrosis after radiotherapy. *Journal of Nuclear Medicine*.

